# Comparative Outcomes of Warfarin vs. Direct Oral Anticoagulants (DOACs) in Anticoagulant-Related Gastrointestinal Bleeding: A Single-Center Study

**DOI:** 10.7759/cureus.74931

**Published:** 2024-12-01

**Authors:** Naqeeb Ullah, Wajeeha Arif, Mohammad B Khan, Heywad T Aioby, Iram Raza, Ambar Siddiq, Urooj Siddiq, Jamil Ahmad, Muhammad Hamza Ghufran, Ayiz Jan, Sundas Safdar, Hanifullah Hanfi, Shahabuddin Zia

**Affiliations:** 1 Internal Medicine, Lady Reading Hospital, Peshawar, PAK; 2 Internal Medicine, Khalifa Gul Nawaz Teaching Hospital, Bannu, PAK; 3 Clinical Sciences, Windsor University School of Medicine, Cayon, KNA; 4 Clinical Research, Windsor University School of Medicine, Cayon, KNA; 5 Internal Medicine, Pakistan Institute of Medical Sciences, Peshawar, PAK; 6 Medicine, Médecins du Monde-France, Pakistan Mission, Peshawar, PAK; 7 Medicine, Lady Reading Hospital, Peshawar, PAK; 8 Medicine, Saidu Medical College, Swat, PAK; 9 Diagnostic Radiology, Lady Reading Hospital, Peshawar, PAK; 10 Medicine, Mardan Medical Complex, Mardan, PAK; 11 Medicine and Surgery, Khyber Medical University, Peshawar, PAK

**Keywords:** anticoagulant therapy, clinical outcomes, emergency management, gastrointestinal bleeding, multidisciplinary care

## Abstract

Background: The management of thromboembolic risk and the necessity for timely hemorrhage control make anticoagulant-related gastrointestinal (GI) bleeding clinically challenging.

Objective: This study aimed to evaluate clinical outcomes (such as bleeding control and mortality) and the effectiveness of anticoagulation reversal techniques in patients with anticoagulant-related GI bleeding in emergency settings.

Methodology: This prospective, observational study conducted at Lady Reading Hospital, Peshawar, from January to December 2023, included patients aged 18 or older with GI bleeding on warfarin or direct oral anticoagulants (DOACs). Key clinical data, including demographics and comorbidities, were collected. The study followed a multidisciplinary approach with emergency physicians, gastroenterologists, and surgeons. Pharmacologic management (Vitamin K for warfarin and idarucizumab for DOACs) was initiated based on clinical judgment, with endoscopic interventions performed within 24 hours if needed, and surgical intervention considered if other methods failed or complications arose. Outcomes such as bleeding control, transfusion needs, and mortality were tracked. Data were collected prospectively via case report forms, and patients were followed for up to six months post-discharge. Statistical analysis was performed using SPSS version 27 (IBM Corp., Armonk, NY), with descriptive statistics for all variables. Continuous variables were compared using independent t-tests, and categorical data were assessed using chi-square tests. Adjusted odds ratios were calculated for mortality and bleeding control outcomes, accounting for confounders.

Results: A total of 384 patients were included, with 180 (46.88%) on warfarin and 204 (53.12%) on DOACs. Bleeding control was significantly better in the DOAC group (170/204, 83.33% vs. 130/180, 72.22%, *P* = 0.03), while mortality was higher in patients on warfarin (20, 11.11% vs. 10, 4.90%, *P* = 0.02). Patients on warfarin also had longer hospital stays (6.89 vs. 5.52 days, *P* = 0.01) and times to intervention (5.28 vs. 4.64 hours, *P* = 0.03). Although demographic characteristics (e.g., age and gender) and comorbidities (e.g., hypertension and diabetes) were comparable between groups, DOACs showed safer profiles during hospitalization. Long-term follow-up outcomes, including readmission rates, recurrent bleeding, and post-discharge mortality, were similar across both groups.

Conclusions: The study demonstrates that DOACs offer better outcomes in bleeding control and mortality compared to warfarin in anticoagulant-related GI bleeding, emphasizing the importance of tailored treatment strategies. These findings highlight the potential benefits of DOACs in emergency settings, supporting the need for customized management plans to optimize patient outcomes.

## Introduction

Anticoagulant medications play a crucial role in the prevention and management of thromboembolic disorders, such as atrial fibrillation, deep vein thrombosis, and pulmonary embolism [1]. While these drugs significantly reduce morbidity and mortality associated with thromboembolic events, they are also a leading cause of bleeding complications, particularly gastrointestinal (GI) bleeding [2]. Managing anticoagulant-related GI bleeding presents a substantial therapeutic challenge, as it requires a careful balance between continuing anticoagulation to prevent thromboembolic events and promptly addressing the hemorrhage [3]. This complexity demands a sophisticated, multidisciplinary approach in emergency settings, encompassing rapid diagnosis, comprehensive risk assessment, and timely therapeutic interventions [4].

In emergency care, anticoagulant-related GI bleeding poses unique challenges due to the high stakes involved in decision-making. For instance, a patient on anticoagulation therapy for atrial fibrillation presenting with GI bleeding must undergo a thorough evaluation to control bleeding effectively without significantly increasing their risk of stroke. This scenario highlights the intricate clinical judgments required in such cases. Furthermore, recent bibliometric analyses of GI bleeding literature, including a comprehensive review of 5,033 articles, underscore advancements in prevention, diagnosis, and management strategies [5]. These studies emphasize the critical role of international collaboration and the influence of high-resource countries in shaping global trends and innovations in GI bleeding management.

The age of the patient, concomitant use of antiplatelet medicines, and pre-existing GI disorders, such as peptic ulcers or varices, may all worsen GI bleeding associated with anticoagulants [6]. Assessment must be completed very once when GI bleeding manifests, since it may vary from subtle to severe hemorrhage [7]. Warfarin has traditionally been the most widely used anticoagulant for managing thromboembolic disorders. However, with the introduction of newer direct oral anticoagulants (DOACs), which have fewer side effects and do not require regular monitoring, clinical recommendations for the treatment of anticoagulant-induced bleeding are evolving [8]. These medicines are not like conventional anticoagulants like warfarin in that they have different mechanisms and need different reversal techniques [9].

For efficient decision-making in emergency settings, a multidisciplinary team is essential. Emergency physicians manage initial stabilization and resuscitation, gastroenterologists perform endoscopic interventions to control bleeding, hematologists oversee anticoagulant reversal and manage coagulation issues, and surgeons handle cases requiring surgical intervention. This coordinated approach ensures timely and effective management of anticoagulant-related GI bleeding [10,11]. Managing anticoagulant-related GI bleeding requires critical interventions such as the timely reversal of anticoagulation (e.g., vitamin K and prothrombin complex concentrate for warfarin and specific antidotes like idarucizumab for DOACs), along with endoscopic examination and other therapeutic treatments [12]. The study aims to evaluate the effectiveness of coordinated care in managing anticoagulant-related GI bleeding in emergency settings. Specifically, it compares the clinical outcomes, bleeding control, mortality rates, and the safety of anticoagulation reversal techniques in patients receiving warfarin versus DOACs. This study emphasizes the role of coordinated multidisciplinary care in improving outcomes for patients with anticoagulant-related GI bleeding.

## Materials and methods

Study design and setting

This prospective, observational study was conducted at Lady Reading Hospital, Peshawar, over one year, from January 2023 to December 2023. The hospital follows established protocols for managing anticoagulant-related GI bleeding, with a multidisciplinary approach involving emergency physicians, gastroenterologists, and surgeons. Pharmacologic interventions, such as the administration of reversal agents (Vitamin K for warfarin and idarucizumab for DOACs), are managed by emergency physicians and hematologists based on clinical parameters, including the patient’s hemodynamic stability and laboratory findings. Endoscopic interventions are performed by gastroenterologists and are typically conducted within 24 hours, depending on the patient’s clinical condition and the need for resuscitation. Surgical intervention is considered only if pharmacologic or endoscopic treatments are ineffective or if complications arise, such as perforation or peritonitis. Decisions regarding surgical intervention are made by a multidisciplinary team, based on the patient’s overall condition and prognosis.

Inclusion and exclusion criteria

**Figure 1:**
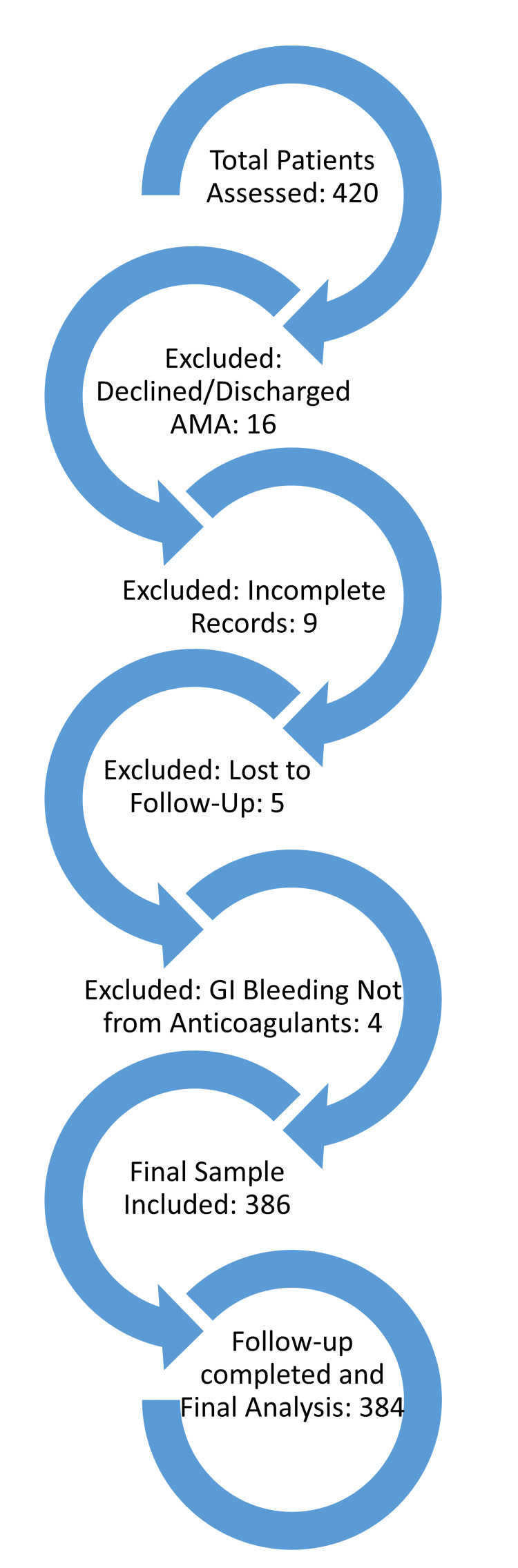
Flowchart of the patient selection process, showing the number of patients assessed for eligibility, excluded based on specific criteria, and included in the final analysis. GI, gastrointestinal; AMA, American Medical Association

The inclusion criteria were patients aged 18 years or older, those receiving anticoagulant therapy (warfarin or DOACs), and individuals requiring emergency care due to GI bleeding. The exclusion criteria included patients who declined treatment, were discharged against medical advice, had incomplete medical records, were lost to follow-up, or presented with GI bleeding unrelated to anticoagulant use. The attribution of GI bleeding to anticoagulant use was based on a systematic approach, including confirmation of anticoagulant therapy through patient history and prescription records, assessment of the temporal relationship between anticoagulant therapy and bleeding onset, exclusion of alternative causes of GI bleeding (e.g., peptic ulcers and varices), and clinical improvement following the administration of reversal agents such as Vitamin K or idarucizumab. Figure 1 shows the flowchart of patient selection, including the number of patients assessed, excluded, and those who were ultimately included in the study.

Sample size

The sample size for this study was calculated using the World Health Organization (WHO) formula for cross-sectional studies: n = Z^2 ^× p × (1 - p)/d^2^ ​, where Z is 1.96 for a 95% confidence level, p is the estimated prevalence (assumed to be 50% for maximum sample size), and d is the desired margin of error (5%). Using these values, the required sample size was approximately 384 patients. This ensures sufficient statistical power to assess multidisciplinary management strategies for anticoagulant-related GI bleeding. 

Data collection

Prospective data collection was carried out on patients presenting to the emergency department with GI bleeding (Appendix). Key clinical factors were systematically recorded, including the patient's clinical presentation, comorbidities, anticoagulant medication, demographic data, and the type of interventions provided (pharmacologic, endoscopic, or surgical). The decision-making process regarding intervention choices was guided by a combination of clinical judgment and operational considerations. For pharmacologic interventions, emergency physicians and hematologists made treatment decisions based on the patient's hemodynamic stability, laboratory results, and the type of anticoagulant therapy used. Reversal agents, such as Vitamin K for warfarin or idarucizumab for DOACs, were administered according to established protocols. Additional medications, like proton pump inhibitors (PPIs) or prokinetic agents, were employed as necessary.

Endoscopic interventions were performed by gastroenterologists and were primarily determined by the patient’s clinical condition, including ongoing bleeding, hemodynamic stability, and relevant laboratory findings, such as hemoglobin levels. If the patient's clinical status allowed, endoscopic intervention was typically performed within 24 hours of presentation, unless immediate resuscitation was required. The decision to proceed with endoscopy was based on the severity of the bleeding and other clinical factors. In cases where pharmacologic or endoscopic treatments were ineffective, or if complications such as perforation or peritonitis developed, surgical intervention was considered. A multidisciplinary team, including gastroenterologists, surgeons, and intensivists, made these decisions based on the patient’s overall condition and prognosis.

The outcomes, including bleeding control, transfusion requirements, and mortality, were carefully documented using structured case report forms. The collected data were stored in a secure computerized database, and patients were followed for up to six months post-discharge. During this follow-up period, outcomes such as recurrence of bleeding and mortality were tracked through outpatient visits, phone interviews, and reviews of electronic health records. Transfusions administered during the study were primarily packed red blood cells (PRBCs), used to manage significant blood loss and anemia. Platelet transfusions, including apheresis thrombocytes, were selectively administered to patients with thrombocytopenia or platelet dysfunction, particularly those with chronic kidney disease (CKD) or coagulopathy related to anticoagulant therapy. Whole blood transfusions were not used in this study, and transfusion decisions were made based on clinical parameters such as hemoglobin levels, platelet counts, and the presence of active bleeding.

Bleeding control

Bleeding control was defined as the cessation of GI bleeding following the initial intervention, which could include pharmacologic, endoscopic, or surgical treatments. For patients receiving pharmacologic treatment, bleeding control was determined by the stabilization of hemoglobin levels, defined as a <2 g/dL drop over 12-24 hours, and the absence of further clinical signs of bleeding, such as hematemesis or melena. Hemoglobin levels were monitored, with a threshold of 7 g/dL considered the standard for assessment. If the hemoglobin remained stable and no additional signs of active bleeding were observed, bleeding was considered controlled. In patients receiving endoscopic or surgical intervention, bleeding control was confirmed by the cessation of visible blood loss during or after the procedure, even if additional interventions, such as transfusions or further pharmacologic treatment, were required. Therefore, bleeding control refers to the cessation of bleeding following the primary intervention but does not exclude the need for further medical or surgical management.

Circumstances of results and definitions

The intervention strategies used in this study were determined by clinical judgment, with an emphasis on stabilizing the patient’s hemodynamic status. Bleeding control was defined as the cessation of GI bleeding following initial intervention, which may include pharmacologic, endoscopic, or surgical treatments. In patients receiving pharmacologic treatment, bleeding control was assessed by monitoring hemoglobin levels, aiming for a <2 g/dL drop over 12-24 hours, and the absence of further signs of active bleeding, such as hematemesis or melena. Endoscopic and surgical interventions were considered when pharmacologic management failed or complications occurred. Bleeding control was confirmed when visible blood loss ceased during or after these interventions, even if additional treatments, such as transfusions, were required.

Statistical analysis

Data analysis was performed using SPSS version 27 (IBM Corp., Armonk, NY). Descriptive statistics were calculated for all variables, including frequencies for categorical data and means with standard deviations (SD) for continuous variables. To compare continuous variables between groups, independent t-tests were used, while chi-square tests were employed for categorical variables (e.g., comparison of outcomes between warfarin and DOAC groups). For assessing the strength of the association between outcomes and treatment types, odds ratios (ORs) with 95% confidence intervals (CIs) were calculated for binary outcomes such as bleeding control, need for transfusion, and mortality. Mean differences were computed for continuous outcomes such as length of hospital stay and time to intervention. A *P*-value of less than 0.05 was considered statistically significant for all tests. For mortality and bleeding control outcomes, adjusted odds ratios (AORs) were calculated where appropriate, adjusting for relevant confounding factors.

Ethical approval

The Institutional Review Board gave their approval to the project. Before being included in the research, all participants or their legal guardians provided written informed permission.

## Results

The main clinical and demographic features of patients with GI bleeding associated with anticoagulants are included in Table 1 and are grouped by type of treatment. It was found that patients on warfarin (mean age 66.42 years) were slightly older than those on DOACs (mean age 64.46 years). The mean age difference between the warfarin and DOAC groups was not statistically significant: mean difference (95% CI): 1.96 (-0.26, 4.18), *t* = 1.177, *P* = 0.235. The gender distribution was similar across both groups, with a slightly higher proportion of males in both warfarin (105 males, 58.33%) and DOAC (115 males, 56.37%) groups. The AOR (95% CI) for the male gender was 1.06 (0.74, 1.51), indicating no significant gender difference between the groups (χ² = 0.179, *P* = 0.673).

**Table 1:** Key demographic and clinical characteristics of patients with anticoagulant-related GI bleeding by therapy type. *P*-values were calculated using independent *t*-tests for continuous variables (age, length of hospital stay, and time to intervention) and chi-square tests for categorical variables (gender, bleeding type, comorbidities, and interventions). *P-value < 0.05 is considered statistically significant. GI, gastrointestinal

Variable		Warfarin (*n* = 180)	DOACs (*n* = 204)	Adjusted OR/mean difference (95% CI)	Test statistics	*P*-value
Age (years)	Mean ± SD	66.42 ± 11.23	64.46 ± 13.05	1.96 (-0.26, 4.18)	*t* = 1.177	0.235
Gender (*n*, %)	Male	105 (58.33)	115 (56.37)	1.06 (0.74, 1.51)	χ² = 0.179	0.673
	Female	75 (41.67)	89 (43.63)	0.94 (0.66, 1.33)		
Type of Bleeding (*n*, %)	Occult	40 (22.22)	60 (29.41)	1.47 (0.83, 2.62)	χ² = 2.47	0.12
	Overt	140 (77.78)	144 (70.59)	0.68 (0.40, 1.15)		
Comorbidities (*n*, %)	Hypertension	102 (56.67)	123 (60.29)	1.22 (0.86, 1.73)	χ² = 0.595	0.448
	Diabetes mellitus	63 (35.00)	71 (34.80)	1.03 (0.69, 1.54)	χ² = 0.003	0.943
	Chronic kidney disease	39 (21.67)	32 (15.69)	1.32 (0.87, 2.00)	χ² = 2.103	0.135
Intervention strategies (*n*, %)	Pharmacologic (reversal agents)	30 (16.67)	35 (17.16)	1.08 (0.67, 1.74)	χ² = 0.187	0.664
	Pharmacologic (managed by emergency physicians and hematologists)	100 (55.56)	120 (58.82)	0.91 (0.63, 1.31)	χ² = 0.293	0.586
	Endoscopic (performed by gastroenterologists)	60 (33.33)	60 (29.41)	1.16 (0.77, 1.74)	χ² = 0.399	0.528
	Surgical (conducted by GI surgeons)	20 (11.11)	24 (11.76)	0.93 (0.52, 1.66)	χ² = 0.042	0.831
Length of hospital stay (days)	Mean ± SD	6.89 ± 3.58	5.52 ± 2.81	1.37 (0.23, 2.51)	*t* = 2.576	0.01*
Time to intervention (hours)	Mean ± SD	5.28 ± 1.59	4.64 ± 1.22	0.64 (-0.11, 1.39)	*t* = 2.114	0.03*

Bleeding patterns also differed: occult bleeding was more common in patients on DOACs (60 patients, 29.41%) compared to warfarin (40 patients, 22.22%), with an AOR (95% CI) of 1.47 (0.83, 2.62). However, this difference was not statistically significant (χ² = 2.47, *P* = 0.12), while overt bleeding was more prevalent in patients on warfarin (140 patients, 77.78%) compared to those on DOACs (144 patients, 70.59%), with an AOR (95% CI) of 0.68 (0.40, 1.15), again showing no statistical significance (χ² = 2.47, *P* = 0.12).

Regarding comorbidities, hypertension was the most common condition in both groups, affecting over half of the patients in each group (102 patients, 56.67% for warfarin and 123 patients, 60.29% for DOACs). The AOR (95% CI) for hypertension was 1.22 (0.86, 1.73), showing no significant difference between the groups (χ² = 0.595, *P* = 0.448). Diabetes mellitus and chronic kidney disease were also prevalent in both groups, though chronic kidney disease was more common in patients on warfarin (39 patients, 21.67%) than in those on DOACs (32 patients, 15.69%), with an AOR (95% CI) of 1.32 (0.87, 2.00) (χ² = 2.103, *P* = 0.135), indicating no significant difference.

In terms of treatment, pharmacologic interventions were the most commonly used strategy, applied to 100 patients on warfarin (55.56%) and 120 patients on DOACs (58.82%). The AOR (95% CI) for pharmacologic intervention was 0.91 (0.63, 1.31), suggesting no significant difference between the groups (χ² = 0.293, *P* = 0.586). Endoscopic interventions were performed in about one-third of patients on warfarin (60 patients, 33.33%) and a slightly lower percentage of patients on DOAC (60 patients, 29.41%), with an AOR (95% CI) of 1.16 (0.77, 1.74) (χ² = 0.399, *P* = 0.528). Surgical intervention was less frequent but still notable, with 20 patients on warfarin (11.11%) and 24 patients on DOAC (11.76%) requiring surgery, with an AOR (95% CI) of 0.93 (0.52, 1.66) (χ² = 0.042, *P* = 0.831), showing no significant difference.

These findings highlight some important differences in clinical presentation, comorbidities, and intervention strategies based on the type of anticoagulant therapy. The mean difference (95% CI) for length of hospital stay was 1.37 (0.23, 2.51) days longer for patients on warfarin (*t* = 2.576, *P* = 0.01), indicating a significant difference, while the mean difference (95% CI) for time to intervention was 0.64 (-0.11, 1.39) hours, showing no significant difference (*t* = 2.114, *P* = 0.03).

The mortality rate was significantly higher in the warfarin group compared to the DOAC group, with 20 patients (11.11%) experiencing total mortality versus 10 patients (4.90%) in the DOAC group (χ² = 5.286, *P* = 0.02). In-hospital mortality was similarly more prevalent among patients on warfarin, with 18 patients (10.00%) affected compared to 7 patients (3.43%) in the DOAC group. Post-discharge mortality, however, was relatively similar between the groups, with 2 patients (1.11%) in the warfarin group and 3 patients (1.47%) in the DOAC group. These findings underscore a notable difference in mortality outcomes, highlighting the increased risk associated with warfarin therapy for patients with GI bleeding.

In terms of bleeding control, a higher proportion of patients on DOAC (170/204, 83.33%) achieved successful bleeding control compared to patients on warfarin (130/180, 72.22%), with a statistically significant difference (χ² = 4.667, *P* = 0.03). Uncontrolled bleeding occurred more frequently in the warfarin group (27.78%) compared to the DOAC group (16.67%). Transfusion requirements were similar between the groups, with 88 patients on warfarin (48.89%) and 90 patients on DOAC (44.12%) requiring transfusions (χ² = 0.656, *P* = 0.42). Collectively, these findings highlight the superior outcomes associated with DOAC therapy in terms of both bleeding control and mortality, underscoring the need for tailored anticoagulant management strategies in patients with GI bleeding.

Bleeding control was significantly better in the DOAC group, with an AOR of 1.74 (95% CI 1.06, 2.85), indicating that patients on DOACs were more likely to achieve controlled bleeding compared to those on warfarin. The need for transfusion did not differ significantly between the groups (AOR: 1.14, 95% CI: 0.77, 1.69), suggesting that both anticoagulants had similar requirements for transfusions.

Regarding mortality outcomes, patients on DOAC had a significantly lower rate of in-hospital mortality (AOR: 3.57, 95% CI: 1.23, 10.26), with patients on warfarin exhibiting a higher risk of death during hospitalization. However, post-discharge mortality showed no significant difference between the two groups (AOR: 0.75, 95% CI: 0.14, 3.98), highlighting that while in-hospital outcomes favor DOACs, long-term survival rates post-discharge are comparable. These adjusted findings provide stronger evidence of the relative safety of DOACs in patients with GI bleeding, particularly concerning bleeding control and in-hospital mortality (Table 2).

**Table 2:** Comparison of clinical outcomes by anticoagulant therapy type. *P*-values were calculated using chi-square tests (*χ*²). *P-value < 0.05 was considered significant. OR, odds ratio

Outcome variable		Warfarin (*n* = 180)	DOACs (*n* = 204)	Adjusted OR/mean difference (95% CI)	χ^2^	*P*-value
Bleeding control (*n*, %)	Controlled	130 (72.22)	170 (83.33)	1.74 (1.06, 2.85)	4.667	0.03*
	Uncontrolled	50 (27.78)	34 (16.67)	-		
	Successful reversal and bleeding control (*n*, %)	50 (27.8%)	60 (29.4%)	1.04 (0.72, 1.49)	0.18	0.67
Need for transfusion (*n*, %)	Transfusion required	88 (48.89)	90 (44.12)	1.14 (0.77, 1.69)	0.656	0.42
	No transfusion required	92 (51.11)	114 (55.88)	-		
Mortality (*n*, %)	Total mortality	20 (11.11)	10 (4.90)	2.43 (1.04, 5.70)	-	-
	In-hospital mortality	18 (10.00)	7 (3.43)	3.57 (1.23, 10.26)	5.286	0.02*
	Post-discharge mortality	2 (1.11)	3 (1.47)	0.75 (0.14, 3.98)		

Table 3 shows no significant difference between the two groups in terms of readmission rates (*P* = 0.48), recurrent bleeding episodes (*P* = 0.35), and post-discharge mortality (*P* = 0.79), suggesting that the anticoagulant type does not significantly impact these outcomes over the long term. Additionally, anticoagulation restart rates were similarly high in both groups (around 94% for warfarin and 90% for DOACs), with thrombotic events post-restart showing no significant difference (*P* = 0.32). This indicates that restarting anticoagulation therapy after GI bleeding did not result in differing rates of thrombotic complications between the two groups.

**Table 3:** Follow-up outcomes at six months post-discharge.

Outcome variable	Warfarin (*n* = 180)	DOACs (*n* = 204)	OR (95% CI)	*χ*²	*P*-value
Readmission rate (%)	25 (13.89%)	22 (10.78%)	1.31 (0.70-2.46)	0.492	0.48
Recurrent bleeding episodes (%)	15 (8.33%)	12 (5.88%)	1.45 (0.65-3.23)	1.076	0.35
Post-discharge mortality (%)	2 (1.11%)	3 (1.47%)	0.76 (0.12-4.74)	0.079	0.79
Anticoagulation restart (%)	170 (94.44%)	185 (90.68%)	1.36 (0.80-2.31)	1.111	0.29
Thrombotic events post-restart (%)	8 (4.44%)	6 (2.94%)	1.52 (0.52-4.44)	0.960	0.32
Bleeding events post-restart (%)	5 (2.78%)	4 (1.96%)	1.44 (0.38-5.34)	0.556	0.46

However, notable differences were observed in terms of bleeding events post-restart, where patients on warfarin had a slightly higher incidence (*P* = 0.46), though this finding was not statistically significant. Despite the overall lack of statistical significance across most outcomes, the OR suggests a slight trend toward greater risks in the warfarin group, particularly for thrombotic events and bleeding complications post-restart. This highlights the importance of individualized anticoagulant management based on patient-specific factors, as DOACs may offer a marginally safer profile in terms of bleeding risk during follow-up. The chi-square tests further reinforce these results, with most outcomes showing no significant differences between the two groups, although the trends could suggest some advantages in terms of safety with DOAC therapy, especially in long-term management after GI bleeding.

## Discussion

Anticoagulant-related GI bleeding presents substantial therapeutic challenges, particularly in balancing the need for anticoagulation to prevent thromboembolic events with the necessity of effective hemorrhage control. With a statistically significant *P*-value of 0.03, we found that 180 (72.22%) patients treated with warfarin and 204 (83.33%) patients on DOACs achieved bleeding control. This result is consistent with other research investigations that found that patients using DOACs exhibited better bleeding control results than those using warfarin. These studies suggested that this was because DOACs provide faster reversal techniques than standard anticoagulants [9,13].

Moreover, our analysis showed that overall mortality was 11.11% for warfarin-using individuals compared to 4.90% for patients on DOACs. This difference, which is backed by a *P*-value of 0.02, emphasizes the elevated risk that comes with taking warfarin medication, which has been substantiated by other research [14]. Patients on warfarin had greater mortality rates and worse bleeding control outcomes compared to those on DOACs, consistent with the findings of Chornenki et al. [15] who noted that warfarin’s variable anticoagulation and higher bleeding risk lead to poorer outcomes. Our study underscores the increased risks of bleeding complications in patients on warfarin, supporting the use of DOACs as a potentially safer alternative for managing anticoagulant-related GI bleeding.

Hospitalization metrics showed that patients on warfarin had a significantly longer average duration of stay compared to those on DOACs, with a *P*-value of 0.01: 6.89 days (SD ± 3.58) versus 5.52 days (SD ± 2.81). This longer stay is consistent with earlier research, which has shown that complications from warfarin administration are often associated with longer hospital admissions [16]. Likewise, with a significant *P*-value of 0.03, patients on warfarin had an average time to the intervention of 5.28 hours (SD ± 1.59) compared to 4.64 hours (SD ± 1.22) for patients on DOACs. This delay in therapy for patients on warfarin emphasizes the need for prompt and efficient treatment regimens and adds to the complexity of management techniques.

Although a nonsignificant *P*-value of 0.42, the need for transfusions was greater among patients on warfarin (88, 48.89%) compared to patients on DOAC (90, 44.12%). This result is in line with other studies and implies that although transfusions are often needed in both groups, differences in the kind of anticoagulant may not have a major effect on the need for transfusions [9,17].

In light of the study's objective to evaluate clinical outcomes and the effectiveness of anticoagulation reversal techniques in anticoagulant-related GI bleeding, our findings emphasize the importance of individualized management strategies. The comparative analysis of Warfarin and DOACs in this context underscores the need for continuous reassessment and refinement of treatment protocols, particularly in emergency settings, to optimize outcomes such as bleeding control and mortality rates. These insights are crucial for improving patient care in settings where anticoagulation therapy and bleeding control need to be carefully balanced, providing a foundation for future research and clinical practice advancements.

Limitations

Several limitations should be considered when interpreting the findings of this study, including potential biases inherent in the observational design, the single-center setting that may limit generalizability to other patient populations or treatment protocols, and the treatment of DOACs as a single group without subgroup analyses, which may mask important differences given their unique pharmacokinetic properties and reversal strategies. Furthermore, while the sample size was calculated for sufficient statistical power, it may not fully capture all variations in patient responses to different anticoagulant treatments, and the relatively short 90-day follow-up period may not account for long-term outcomes in this cohort. To address these limitations, standardized data collection and multivariable statistical adjustments were employed to control for confounding factors, and the inclusion of a diverse patient population alongside comparisons with other studies aimed to enhance external validity. Detailed data on individual DOAC agents were collected to facilitate future subgroup analyses, and sensitivity and stratified analyses were performed to ensure the robustness of the findings despite sample size constraints. Additionally, a rigorous follow-up protocol involving outpatient visits, phone interviews, and electronic health record reviews was implemented to ensure comprehensive tracking of outcomes, thereby minimizing potential biases and enhancing the reliability and applicability of the results.

## Conclusions

This study highlights significant differences in clinical outcomes between patients treated with warfarin and those treated with DOACs for anticoagulant-related GI bleeding. Our results show that patients on DOAC had a significantly higher rate of bleeding control (204, 83.33%) compared to patients on warfarin (130, 72.22%), with a statistically significant difference (*P* = 0.03). In addition, patients on DOACs had lower mortality rates, with 10 (4.90%) compared to 20 (11.11%) in the warfarin group (*P *= 0.02). In-hospital mortality was also significantly lower in the DOAC group (7, 3.43%) compared to the warfarin group (18, 10.00%). Furthermore, patients on DOAC had shorter hospital stays (mean 5.52 days) compared to patients on warfarin (mean 6.89 days), with a statistically significant difference (*P* = 0.01). These findings underscore the potential advantages of DOACs in improving clinical outcomes, particularly in terms of bleeding control, mortality, and hospital stay duration.

However, while these results are promising, our study also has limitations. One important limitation is the treatment of DOACs as a single group without performing subgroup analyses of individual medications (e.g., apixaban, rivaroxaban, dabigatran, and edoxaban). Given the distinct pharmacokinetic properties and reversal strategies for each DOAC, future studies should investigate these drugs separately to better understand their individual effects on bleeding control, mortality, and other clinical outcomes. Additionally, while our sample size provided sufficient statistical power, further research with larger, multicenter cohorts and longer follow-up periods would be beneficial to validate these findings and explore long-term outcomes. Tailored management strategies, informed by the specific anticoagulant used, are essential for optimizing patient outcomes and improving the effectiveness of multidisciplinary emergency care.

## Appendices

Appendix 

**Table 4:** Structured case report forms for outcomes anticoagulant-related gastrointestinal bleeding.

Variable	Options				Patient Response
Patient ID					Write ID number
Age (years)	< 18	18-30	31-40	> 40	Select Age
Gender	Male	Female	Other		Select Gender
Comorbidities	Hypertension	Diabetes	COPD	Cancer	Specify Comorbidities
GI Bleeding Symptoms	Hematemesis	Melena	Hematochezia	Abdominal Pain	Select Symptoms
Hospital Admission Date					Select Date
Type of Anticoagulant	Warfarin	DOACs	None		Specify Type
INR Level (for Warfarin)	< 1.5	1.5-2.0	2.1-3.0	> 3.0	Select INR Level
Last Dose of DOAC (for DOAC users)	< 12 hrs	12-24 hrs	> 24 hrs		Select Time
Date of Anticoagulant Administration					Select Date
Pharmacologic Treatment	PPIs	Prokinetic Agents	Anticoagulant Reversal (Vitamin K)	Other Specify	Select Medication
Reversal Agent Given	Yes	No			Select Yes/No
Type of Reversal Agent	Vitamin K	Idarucizumab	Andexanet Alpha	Other Specify	Select Type of Agent
Endoscopy Performed	Yes	No			Select Yes/No
Endoscopy Timing	Within 6 hrs	Within 12 hrs	Within 24 hrs	Not Applicable	Select Timing
Surgical Intervention	Yes	No			Select Yes/No
Hemoglobin Level (Initial)	< 7 g/dL	7-10 g/dL	10-12 g/dL	> 12 g/dL	Select Hemoglobin Level
Bleeding Control	Yes	No			Select Yes/No
Transfusion Required	Yes	No			Select Yes/No
30-Day Mortality	Yes	No			Select Yes/No
Follow-Up Date (Days Post Discharge)	< 30 days	30-60 days	> 60 days		Select Follow-Up Duration
Recurrence of Bleeding	Yes	No			Select Yes/No

## References

[1] Diavati S, Sagris M, Terentes-Printzios D, Vlachopoulos C (2022). Anticoagulation treatment in venous thromboembolism: options and optimal duration. Curr Pharm Des.

[2] Lanas-Gimeno A, Lanas A (2017). Risk of gastrointestinal bleeding during anticoagulant treatment. Expert Opin Drug Saf.

[3] Hylek EM (2003). Complications of oral anticoagulant therapy: bleeding and nonbleeding, rates and risk factors. Semin Vasc Med.

[4] Dager WE, Trujillo TC, Gilbert BW (2023). Approaches to precision-based anticoagulation management in the critically Ill. Pharmacotherapy.

[5] Kudu E, Danış F (2022). The evolution of gastrointestinal bleeding: a holistic investigation of global outputs with bibliometric analysis. Turk J Gastroenterol.

[6] Abraham NS (2016). Prevention of gastrointestinal bleeding in patients receiving direct oral anticoagulants. Am J Gastroenterol Suppl.

[7] Kim BS, Li BT, Engel A, Samra JS, Clarke S, Norton ID, Li AE (2014). Diagnosis of gastrointestinal bleeding: a practical guide for clinicians. World J Gastrointest Pathophysiol.

[8] Crawley RM, Anderson RL (2020). Prevention and treatment of bleeding with direct oral anticoagulants. Drugs.

[9] Milling TJ, Refaai MA, Sengupta N (2021). Anticoagulant reversal in gastrointestinal bleeding: review of treatment guidelines. Dig Dis Sci.

[10] Gibler WB, Racadio JM, Hirsch AL, Roat TW (2019). Management of severe bleeding in patients treated with oral anticoagulants: proceedings monograph from the emergency medicine cardiac research and education group-international multidisciplinary severe bleeding consensus panel. Crit Pathw Cardiol.

[11] Martin AC, Benamouzig R, Gouin-Thibault I, Schmidt J (2023). Management of gastrointestinal bleeding and resumption of oral anticoagulant therapy in patients with atrial fibrillation: a multidisciplinary discussion. Am J Cardiovasc Drugs.

[12] Backus B, Beyer-Westendorf J, Body R (2023). Management of major bleeding for anticoagulated patients in the emergency department: an European experts consensus statement. Eur J Emerg Med.

[13] Kaide CG, Gulseth MP (2020). Current strategies for the management of bleeding associated with direct oral anticoagulants and a review of investigational reversal agents. J Emerg Med.

[14] Little DH, Sutradhar R, Cerasuolo JO (2021). Rates of rebleeding, thrombosis and mortality associated with resumption of anticoagulant therapy after anticoagulant-related bleeding. CMAJ.

[15] Chornenki NL, Tritschler T, Stucki F (2022). All-cause mortality after major gastrointestinal bleeding among patients receiving direct oral anticoagulants: a protocol for a systematic review and meta-analysis. Syst Rev.

[16] Tapaskar N, Ham SA, Micic D, Sengupta N (2022). Restarting warfarin vs direct oral anticoagulants after major gastrointestinal bleeding and associated outcomes in atrial fibrillation: a cohort study. Clin Gastroenterol Hepatol.

[17] Di Minno A, Spadarella G, Spadarella E, Tremoli E, Di Minno G (2015). Gastrointestinal bleeding in patients receiving oral anticoagulation: current treatment and pharmacological perspectives. Thromb Res.

